# Polymer Inclusion Membranes (PIMs) for Metal Separation—Toward Environmentally Friendly Production and Applications

**DOI:** 10.3390/polym17060725

**Published:** 2025-03-10

**Authors:** Marin Senila

**Affiliations:** INCDO-INOE 2000, Research Institute for Analytical Instrumentation, 67 Donath Street, 400293 Cluj-Napoca, Romania; marin.senila@icia.ro

**Keywords:** polymer inclusion membrane, metal extraction, sample pretreatment, passive sampling, metal recovery, water treatment, membrane extraction, green extraction

## Abstract

Polymer inclusion membranes (PIMs) have been reported to be useful for the selective separation of numerous metal ions, with multiple applications in areas such as analytical chemistry, water quality monitoring, water treatment, and metal recovery. This review aims to update the recent advancements related to PIM technology in metal ion separation, with a particular emphasis on environmentally friendly production and applications. PIMs have many advantages over classical liquid–liquid extraction, such as excellent selectivity, ease of use with simultaneous extraction and back-extraction, stability, and reusability. PIMs typically consist of a base polymer, a carrier, and, if necessary, a plasticizer, and can therefore be tailored to specific analytes and specific matrices. Consequently, numerous studies have been carried out to develop PIMs for specific applications. In analytical chemistry, PIMs have been used mostly for analyte preconcentration, matrix separation, speciation analysis, and sensing. They can be used as passive sampling tools or integrated into automated water monitoring systems. PIMs are also widely studied for the extraction and purification of valuable metals in the frame of the circular economy, as well as for wastewater treatment. Even if they are a greener alternative to classical metal extraction, their production still requires petroleum-based polymers and toxic and volatile solvents. In recent years, there has been a clear trend to replace classical polymers with biodegradable and bio-sourced polymers and to replace the production of PIMs using toxic solvents with those based on green solvents or without solvents. According to the published literature, environmentally friendly PIM-based techniques are a highly recommended area of future research for metal ion separation directed toward a wide range of applications.

## 1. Introduction

Metals and metalloids are ubiquitous and non-biodegradable elements present in all areas of the environment, having entered through either natural or anthropogenic pathways [1,2,3]. Because only a few metals in the periodic table are essential for living organisms, and their concentration is decisive in determining their toxicity, it is very important to carefully monitor these and apply decontamination measures if the maximum permitted levels are exceeded [4,5]. Moreover, in the paradigm of the circular bioeconomy, valuable metals can be recovered from industrial effluents and reused [6]. In these contexts, analytical methodologies are continuously improved to ensure their high sensitivity and selectivity for metal determination [7], and, in the water treatment field, the facilities for the removal of metal ions are continuously technologically enhanced. As part of these efforts, in the last decades, the concept of polymer liquid membranes has been proposed as an alternative to classical solvent extraction, ion exchange, adsorption, or precipitation techniques [8,9].

Polymer inclusion membranes (PIMs) are a type of liquid membrane designed to eliminate several disadvantages such as inadequate mechanical stability and the risk of carrier leakage. PIMs are designed by embedding the liquid phase in a solid matrix, which increases the stability of the membrane and extends its lifetime, improving its viability for industrial applications [10]. The production of Solvent Polymeric Membranes (SPMs) was first reported by Bloch et al. [11]. These were prepared by pouring a carrier polymer on paper, which enhanced its mechanical and diffusion properties. Later, Sugiura [12] upgraded this material by including a plasticizer in the membrane composition, which improved the mechanical strength properties of the polymer film, meaning paper was no longer necessary [13]. PIMs are characterized by their ability to facilitate the selective transport of metal ions through a polymeric matrix, which can be tailored by incorporating various carriers and plasticizers. Research has shown that, compared to traditional solvent extraction methods, PIMs containing ionic liquids or surfactants as carriers exhibit enhanced selectivity for specific metal ions [14]. PIMs consist of a base polymer that offers structure and mechanical support to the membrane, an extractant (carrier), and, if required, a plasticizer or modifier [15]. To obtain homogeneous and flexible PIMs, the base polymers’ and carriers’ compatibility is vital, and these should be soluble in the same solvent. The solvent’s nature and quantity can influence PIMs’ performance and quality by affecting the dispersion of the carriers and base polymers [10].

PIMs represent an important advance in membrane separation technology, mainly for metal separation. PIMs have transformed traditional separation techniques through their outstanding selectivity, cost-effectiveness, and versatility and by introducing the possibility of simultaneously performing extraction and stripping in one operation [9]. Thus, PIMs are positioned as a sustainable choice for metal separation in analytical chemistry, water treatment, and metal recovery due to their selectivity, recyclability, and ease of use. Despite the advantages of PIMs, their production involves the use of base polymers such as poly (vinyl chloride) (PVC), cellulose triacetate (CTA), or poly (vinylidene fluoride-co-hexafluoropropylene) (PVDF HFP), which are known to be non-biodegradable and are converted into micro- and nanoplastics, causing environmental issues. Moreover, the typical casting method for preparing PIMs involves dissolving all components (base polymer, carrier, and plasticizer) into a volume of a suitable solvent such as tetrahydrofuran, chloroform, or dichloromethane. This mixture is then cast into a mold, and the solvent evaporates slowly [16], which may lead to environmental and health issues. Consequently, it is of great interest to replace the classical chemicals used in PIM preparation with sustainable and greener alternatives. Since several previous reviews presented PIMs’ functioning principles, components, and applications [6,8,9,10,15,17,18,19], this paper aimed to present a thorough overview of the recent research on the advances in PIM applications related to the aqueous solutions for metal separation, with a special focus on sustainable and environmentally friendly materials and methods used to produce PIMs.

## 2. Methodology

The academic databases Clarivate, Scopus, and Google Scholar were used to search publications linked to PIMs. The search keywords “polymer inclusion membranes” and “metal” were used in the first step. Other keywords were “PIM-based metal extraction”, “PIM-based passive sampling”, “PIM-based metal separation”, “green membrane preparation”, and “water purification”. Only papers addressing the use of PIMs for metal separation were considered appropriate to be incorporated in this review. Papers written in English were considered the most relevant and were shortlisted for the review.

## 3. Functioning Principle of Polymer Inclusion Membranes

**Figure 1:**
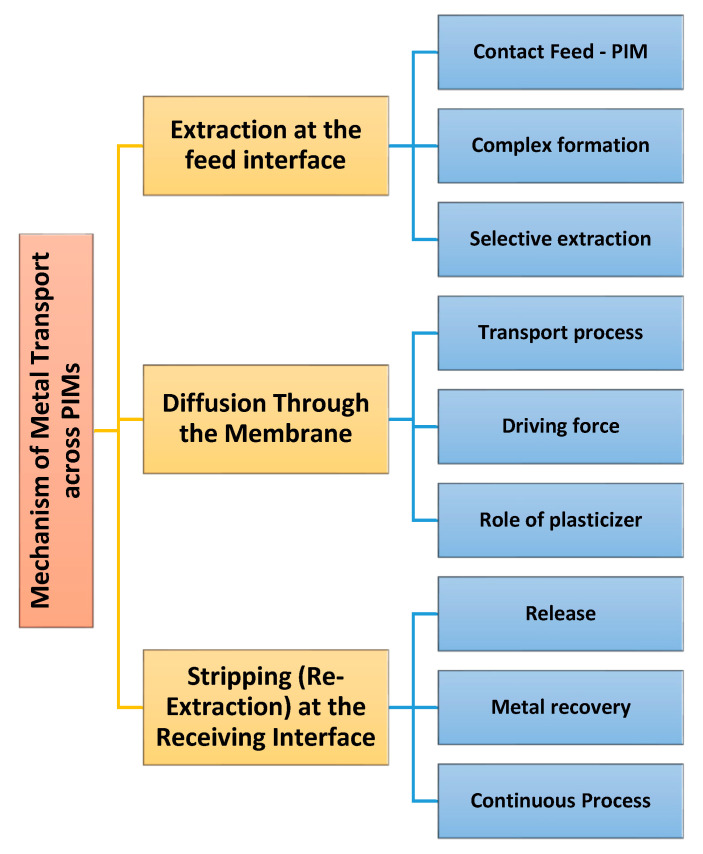
The mechanism of metal ions passing through a PIM.

As stated in the Introduction, the components of PIMs are a base polymer, a carrier, and a plasticizer/modifier. Understanding the functioning principles and transport mechanisms for PIMs is a key element of appraising their efficiency in selective extraction processes. The principal transport mechanism for metals through PIMs is based on carrier-mediated diffusion [9]. The mechanism of metal ions passing through a PIM usually occurs via the steps shown in Figure 1.

Typically, PIMs have been used to separate two aqueous media, whereas ions were transported through the membrane. As presented in Figure 1, the transport of metal ions across the PIM usually occurs via three main steps [6,10]. The first step consists of the extraction of metal ions at the feed interface. Therefore, the PIM is placed in contact with a feed solution containing the metal ions to be extracted. The carrier molecules interact with it and bind the target ions, making a metal–carrier complex at the feed solution–membrane interface. The selectivity depends on the chemical affinity of the carrier, and thus only specific metal ions are extracted into the membrane phase [6]. The second step is represented by the diffusion through the membrane. The developed metal–carrier complexes diffuse through the polymer matrix due to a concentration gradient between the feed solution and the receiving side of the membrane. In general, this transport follows Fick’s law of diffusion [9,20], represented in Equation (1):

(1) $$J=-D\frac{\delta y}{𝜕x}$$

where *J* is the flux, *D* is the metal ion diffusion coefficient across the membrane, and $\frac{𝜕y}{𝜕x}$ is the gradient of concentration. However, this model does not satisfactorily account for the effect of carriers in the transport through PIMs, since these may increase the transport efficiency. Thus, mathematical models analogous to those related to the reaction kinetics have been developed to define the transport across membrane systems considering the experimental data of metal ions transported by several carriers, and the volume of the feed and stripping solutions [21].

Generally, the plasticizer improves the fluidity of the membrane and assists in the faster and more efficient diffusion of the metal–carrier complexes. In the third step, the metal ions are stripped (re-released) at the receiving interface. Upon arrival at the interface with the receiving solution, physicochemical conditions such as pH, ionic strength, or the existence of a stripping agent aid the dissociation of the metal–carrier complexes. The metal ions are released into the receiving phase, and the carrier is regenerated inside the membrane; thus, it can participate in a new extraction process [18]. This operation is cyclic and enables the continuous extraction and separation of metals and their preconcentration [22].

**Figure 2:**
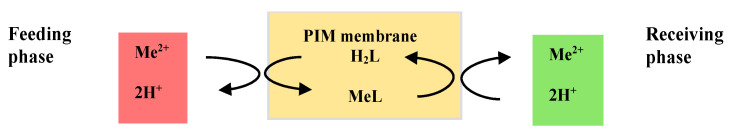
Metal transport across PIMs.

Very importantly, the transport of the analyte through the PIM occurs even if the analyte concentration in the receiving phase becomes higher than in the feed phase, explaining its potential use for analyte preconcentration. An example of the transport process of a bivalent metal Me(II) through a PIM containing an acidic diprotic carrier (H2L) and a mineral acid as a stripping reagent is schematically illustrated in Equation (2) and Figure 2 [23].

(2) $${Me}^{2+}+H_{2}L⇆MeL+2H^{+}$$

**Figure 3:**
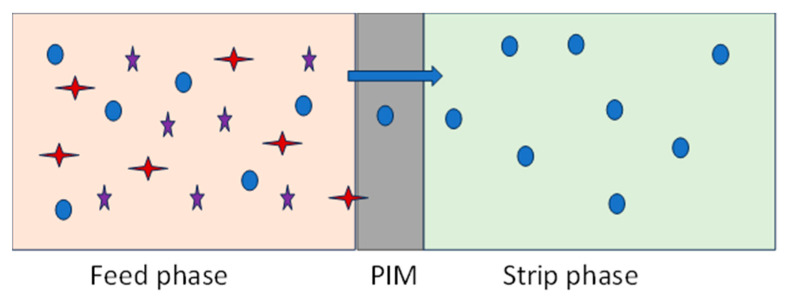
Schematic representation of the selective transport of a specific metal ion from the feed phase, through PIMs, to the strip phase (based on [6,22]).

The carrier accomplishes the metal ion extraction from the aqueous media and their transfer inside the membrane assembly. Therefore, the carrier characteristics are significant, since they affect the process of the metal’s transport. Depending on the carrier class, metal ions are transported through the membrane in several ways: (*i*) simple transport due to metal solubility in the liquid membrane; (*ii*) supported transport caused by partitioning, complexation, or diffusion reaction; (*iii*) counter-transport, produced by the concentration gradient; or (*iv*) co-transport, which implies that a liquid substance is co-transported with an associated component in a process that ends when the concentrations between the receiving phase and the feeding phase are equal [6]. Figure 3 presents a simple representation of the selective separation of a metal ion species from the feed solution, which contains a complex matrix of other chemical species, to the strip phase, through a PIM at their interface [6,22].

Most frequently, the metal flux is calculated based on equations derived from Fick’s first law, considering that the concentration of the complex is negligible at the membrane-receiving phase interface [24]. The flux *J* can be correlated with the variations in metal ion concentration, the volume (*V*) of the feed solution, and the area (*A*) of the exposed membrane (cm^2^), using Equation (3) [25]:

(3) $$J=-\frac{dCV}{dtA}$$

The integrated flux can be accounted for using Equation (4):

(4) $$ln\frac{C}{C_{0}}=-k\cdot t$$

where *C* is the metal ion concentration (mg/L) in the donor solution at time *t*, *C*_0_ is the initial metal ion concentration (mg/L), *k* represents the rate constant (s^−1^), and *t* represents the extraction time (s) [25].

The permeability coefficient (*P*) (cm s^−1^) can be calculated using Equation (5) [26]:

(5) $$P=\frac{V}{A}k$$

Using the permeability coefficient, the initial flux (*J_i_*) can be calculated (Equation (6)):

(6) $$J_{i}=P\cdot C_{i}$$

Nevertheless, in some cases, the experimental results show that the dependency of ln(c/c0) versus time is not always linear, and some authors recommend modified equations to explain the transport of metal ions. For instance, Szczepański [27] used two kinetic models to improve the description of the transport kinetics, which were fitted to the experimental results of Zn(II), Cd(II), Cu(II), and Pb(II) transport through PIMs produced from CTA (base polymer), D2EHPA (carrier), and NPOE (plasticizer). The proposed model, based on an equation such as the first-order chemical reaction equation with equilibrium, provided an improved nonlinear fit to the experimental data and more accurately estimated values of the initial maximum fluxes and permeation coefficients. In a later study, Szczepański [28] compared five mathematical models to describe the transport of Zn(II), Cd(II), Pb(II), and Cu(II) in PIMs prepared with different carriers (tri-n-octylphosphine oxide (TOPO), trihexyl(tetradecyl)phosphonium chloride (Cyphos IL 101), di-(2-ethylhexyl) phosphoric acid (D2EHPA), methyl trioctyl ammonium chloride (Aliquat336), and 3-(1,3-diethoxy-1,3-dioxopropan-2-yl)-1-octylimidazolium bromide (RILC8_Br)). These carriers were mixed in different ratios with CTA (base polymer) and NPOE (plasticizer). A comparison of the initial maximum transport fluxes of Zn(II), Cd(II), Pb(II), and Cu(II) obtained with different carriers is presented in Table 1 [21]. As can be observed, the carrier type strongly influences the initial maximum fluxes.

Temperature is a parameter affecting the transport process. The activation energy of the transport process can be calculated at various temperatures using Equation (7) [26]:

(7) $$lnk=\ln\left(A\right)-\frac{E_{a}}{R}(\frac{1}{T})$$

where *k* is the reaction rate constant, *A* is the pre-exponential factor, *Ea* represents the activation energy, *R* is the gas constant, and *T* is the absolute temperature. The activation energy calculation is important to assess if the transport of a metal ion is a process controlled by diffusion.

In the context of PIM studies, it is important to ascertain the occurrence of interactions at the interfaces and to determine whether these interactions are regular or not in terms of thermodynamics. Consequently, in addition to calculating the activation energy, the thermodynamic parameters, namely the activation entropy change (ΔS^#^) and activation enthalpy change (ΔH^#^), should be calculated using the Eyring equation (Equation (8)):

(8) $$ln\frac{k}{T}=ln\frac{k_{B}}{h}+\frac{{∆S}^{\#}}{R}-\frac{{∆H}^{\#}}{R}(\frac{1}{T})$$

where *T* is the absolute temperature, *k* is the reaction rate constant, *R* means the gas constant, *h* is Planck’s constant, and *k_B_* is the Boltzmann constant.

*ΔS*^#^ and *ΔH*^#^ can be established from the kinetic dataset achieved from a ln *k*/*T* against 1/*T* plot. [26,29].

## 4. Applications of Polymer Inclusion Membranes in Metal Separation

**Figure 4:**
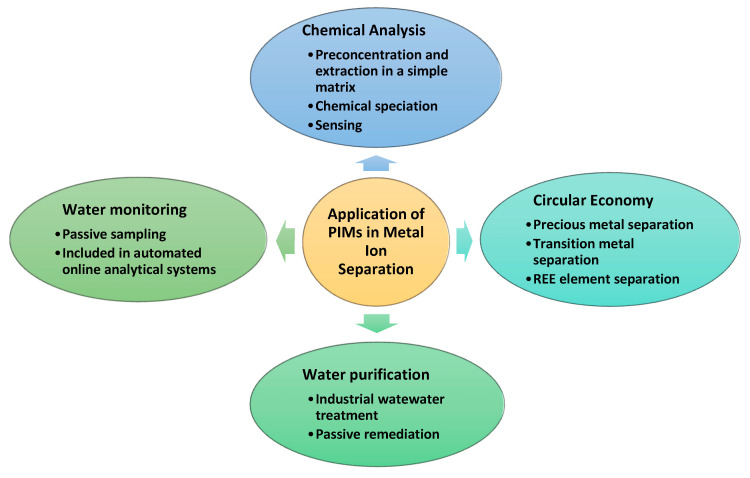
Applications of PIMs in metal ion separation.

PIMs are physically and chemically versatile since their composition can be tailored for different media and target metal ions by selecting appropriate polymers, carriers, and plasticizers [22]. Due to their excellent properties, PIMs can be used for many applications in metal ion separation, the most important being summarized in Figure 4.

### 4.1. Chemical Analysis

Due to the PIM’s ability to perform extraction and back-extraction at the same time, the quantity of solvents used for sample preparation is significantly reduced. Moreover, PIMs can be tailored for the selective extraction of specific analytes, making them superior compared to conventional methods such as solvent extraction and ion exchange and suitable for the separation of target analyte from complex matrices [15]. Accordingly, PIMs have found many applications in chemical analysis, including analyte preconcentration, analyte extraction from a complex to a simple matrix, speciation analysis, and sensing. PIMs can thus improve the limit of detection (LOD) and limit of quantification (LOQ) if deployed for a long period of time. Also, the extraction of analyte in a simple matrix reduces the matrix and spectral interferences in metal determination using a spectrometric method. Due to their excellent specificity, the PIMs are increasingly used to improve chemical speciation analysis by separating species of certain elements with different toxicities [30]. However, the first applications of PIMs in analytical chemistry were as components in optodes and ion-selective electrodes (ISEs), for sensing [22]. Table 2 summarizes several recent examples of PIM application in metal ion analysis.

The advantages of PIMs have been used to develop environmentally friendly and cost-effective methods to address metal or metalloid speciation in water samples. A PIM containing CTA as base polymer and Cyanex 301 (bis(2,4,4-trime thylpentyl)dithiophosphinic acid) as an extractant was recently used for As(III) extraction and preconcentration from water, which allowed direct determination using energy-dispersive X-ray fluorescence (EDXRF) spectrometry [31]. Fontas et al. [32] developed a PIM-based methodology that allows the determination of both arsenate and arsenite species in water. A PIM prepared from PVC and Aliquat 336 as a carrier was reported to transport only As(V), whereas if As(III) is oxidized, total As (in the form of As(V)) is transported and preconcentrated. A PIM obtained from 50% Aliquat 336 as a carrier and 50% CTA as a polymer was tested for the separation of As(III) and As(V) species [33]. An effective separation of As(V) from As(III) was obtained within 5 h (99.7% separation efficiency). Also, the preconcentration allowed the determination of As by atomic absorption spectroscopy, which is a less sensitive technique than inductively coupled plasma mass spectrometry (ICP-MS). Moreover, the authors stated that this is a cost-effective method, estimating a production cost of about USD 0.08–0.16 per 1 m^2^ of PIM.

PIMs were also reported as means for analyte separation from complex matrices, to eliminate matrix effects. Govindappa et al. [34] prepared a low-cost membrane from recycled PVC and benzalkonium chloride (BAC) as a carrier. A PIM containing 50% PVC/40% BAC/10% DOP displayed the maximum transport efficiency for As(V). Macías et al. [36] prepared two types of PIMs, one with D2EHPA and another with Aliquat 336, as carriers for the extraction and preconcentration of Pb(II), Cd(II), and Zn(II) from seawater. A PIM composed of PVC + D2EHPA was tested for the measurement of free Zn(II) concentration in a nutrient solution, as a tool for the assessment of bioavailable species. Alcalde et al. [39] prepared PIMs using CTA, D2EHPA, and NPOE in different compositions. Experiments were carried out to test the effect of metal concentration and ligands on metal accumulation. The presence of ligands (ethylenediamine tetraacetic acid (EDTA) or humic acids) decreased metal accumulation. This indicates that PIM has the ability to differentiate between free metal ions and complexed metal species and can therefore be used in bioavailability studies.

Optodes are a type of chemical sensor that transforms the concentration of the target analyte into an analytical signal generated by an interaction between an active component of the optode and an analyte. These modifications are observed using spectral optical instruments [19]. As reported in the recent literature, PIMs-based optodes have evolved in terms of sensitivity, using optical spectrometric detectors to measure fluorescence, absorbance, or reflectance. The simplest experimental design using a PIM-based optode involves deploying it in the analyzed solution, followed by the determination of the optical properties of the PIM. Also, optodes can be combined with flow-based techniques [18]. A PIM produced from CTA as the base polymer, Kelex 100 as the carrier, and NPOE as the plasticizer was used for sensing Cd(II) in water by in situ visible and mid-Fourier transform infrared spectroscopy analyses [40]. The authors reported comparable results obtained using the new PIM-based sensor and flame atomic absorption spectrometry (FAAS) for real water sample analysis.

### 4.2. Passive Sampling and Water Quality Monitoring

The assessment of the toxic elements in aquatic systems has become a key topic of global concern [41]. Currently, there is an increasing interest in finding easy-to-use and inexpensive alternatives to grab sampling for better water quality monitoring. Passive sampling (PS) is a technique which accounts for the free uptake of analytes from the analyzed sample to a receiving phase within the sampling device, due to the gradient of concentration of the analyte [19,42,43]. There is no active transport or pumping used for these samplers. The analytes pass through a diffusion-limiting membrane without active transport or pumping [20]. A summary of relevant surveys concerning the use of PIMs as passive samplers for water quality assessment and monitoring is presented in Table 3.

Almeida et al. [23] tested two designs of laboratory-scale passive samplers, in which a membrane made from 60% PVC and 40% D2EHPA was included. In the first setup, the passive sampler was immersed in the source solution for a known deployment period. In the second approach, the feed solution was flown through the membrane (“flow-through” approach). The latter method was found to be more appropriate for the calibration of the PIM-based passive sampler. The authors concluded that the PIM-based PS can be applied for Zn monitoring in various aquatic systems. A laboratory experiment was performed to evaluate the prediction capacity of a PIM sampler made from CTA (base) + TEHP (plasticizer) + Kelex-100 (carrier) for Cu(II) passive sampling under different conditions of pH, temperature, metal concentration, flow velocity, and ionic strength. The concentration of Cu ions estimated from the PIM sampler was comparable to that obtained by direct measurements of the solution, suggesting that PIMs can be robust tools when used as passive samplers [44].

A PS consisting of a polytetrafluoroethylene (PTFE) chamber and a PVC membrane with D2EHPA as a carrier was produced and optimized for the detection of Cu, Ni, Co, and Cd in surface waters. The developed sampler proved a long retention time (up to 5 days) followed by linear uptake of analytes for up to 12 days. The order of separation of the analytes was Cd >> Cu >> Co > Ni [45]. Nitti et al. [46] developed a portable system based on a flow-through passive sampler (FTPS) to eliminate the major cations (Na^+^, K^+^, Ca^2+^, Mg^2+^) that compete with Zn^2+^ in extraction. The used PIMs in the system contained dinonylnaphthalene sulfonic acid (DNNS) or D2EHPA as extractants and 1-tetradecanol as a modifier. Experiments were carried out to assess the effects of pH, competing cations, and temperature on Zn^2+^ accumulation in the receiving solution. In general, due to their selectivity and sensitivity, PIMs remain of interest for future developments in PS tools.

### 4.3. Water Purification

Membrane processes represent an attractive alternative to conventional approaches of metal ion removal from wastewater. These have a high efficiency and low energy consumption and can be used at ambient temperatures [47]. Efforts have been made in the last years to produce new PIMs with improved removal efficiency. A summary of the recent literature on water purification using PIMs as separation tools is presented in Table 4.

PIMs were found to have high efficiency in the removal of toxic elements from highly loaded and difficult-to-clean wastewaters resulting from metallurgic or mining industries. Kaya et al. [26] prepared a calixarene-based PIM and tested it for the elimination of chrome plating bathwater of Cr(VI). The membrane was prepared from *p*-*tert*-butylcalix[4]arene amine derivative as an ion carrier, CTA as the base polymer, and 2-NPOE as the plasticizer. The authors reported a transport efficiency using the PIM of approximately 97.69% and showed high selectivity in eliminating Cr(VI) from wastewater. Sellami and collaborators [48,49] developed a new PIM for Cr(VI) separation from polluted water. This contained base polymer poly(vinylidene fluoride) (PVDF), Aliquat 336 as the carrier, and native sodium and organo-modified montmorillonites.

A PIM consisting of PVC as the polymer and 3-propyl-pentane-2,4-dione as the carrier was applied to remove Cr(III), Zn(II), and Ni(II) from post-galvanic wastewater [50]. A CTA-based polymer with Cyanex 921 carrier was tested for As(V) removal from aqueous leachates [51]. PIM selectively removed As(V) with an efficiency of 90%. For Hg(II) separation, a PIM containing a calix[4]pyrrole derivative (carrier) was developed. The separation efficacy was up to 91.8% [41]. A PIM composed of CTA, Kelex 100 as extractant, and tris(2 ethylhexyl) phosphate (TEHP) as the plasticizer was tested for Ni(II) transport [52], whereas a PIM containing multi-walled carbon nanotubes was tested for Zn(II) removal from an aqueous solution. Konczyk and Ciesielski [55] prepared a CTA-based PIM in which calixresorcin[4]arene derivates were tested for the transport of Pb(II) ions.

Maiphetlho et al. [54] reported the use of PIM as a tool for passive remediation of AMD via the retention of metal ions (Cu, Cd, Co, Ni, and Fe) at an acidic pH (3.21). The authors reported that the PIM can efficiently remove toxic metals even at low pH levels. Eyupoglu and Unal [56], in a recently published paper, reported the use of PVDF-co-HPF combined with different plasticizers, and ionic liquids to produce PIMs for the removal of Cd(II) from aqueous solutions. A maximum mass transfer rate of 1.26 μmol s^−1^ m^−2^ was reported.

PIMs’ practical utility under real wastewater conditions is contingent upon their resistance to biofouling and long-term stability. Biofouling, which means the accumulation of microorganisms on the PIM surface, may pose significant challenges. Studies revealed that one of the reasons for biofouling is the presence of nutrients in the feed water, and that it also depends on the nutrient concentrations. For instance, AlSawaftah et al. [57] reported that biofilms grown with lower phosphorus concentrations (3 μg/L) can be easily removed hydraulically, compared to those grown at a concentration of 6 μg/L. Thus, controlling nutrient levels may offer an approach to lessen biofouling. To improve biofouling resistance, several modifications to PIMs have been explored. The incorporation of nanoparticles into the polymer matrix has been shown to decrease fouling and improve PIMs’ performance [58]. Similarly, the addition of graphene oxide and vanillin to the polymer matrix significantly increased fouling resistance [59]. These adjustments suggest possible pathways to improve the biofouling resistance of PIMs.

On the other hand, PIMs have been reported to be resistant to biofouling, because some of their components, such as Aliquat336, which is a combination of quaternary ammonium, act as antibacterial and antifungal agents [33,60]. Furthermore, cleaning processes intended to eliminate fouling can involuntarily contribute to PIM degradation. For instance, repetitive backwashing can lead to the degradation of the membrane material [61]. Thus, while developing PIMs, it is crucial to study not only their initial performance but also how they endure functioning stresses and cleaning protocols over prolonged periods.

### 4.4. Circular Economy

The recovery of valuable metals in the circular economy paradigm is continuously increasing due to their demand in industrial applications. Their recovery using pyrometallurgical or hydrometallurgical processes poses a threat to the environment [62]. Given their excellent selectivity, PIMs represent eco-friendly and easy-to-use solutions for the extraction of valuable metals. Recent studies have focused on the development of new technologies for the recovery of metal ions from industrial wastes as secondary sources. A selection of the recent literature linked to the recovery of valuable metals from waste using PIMs is presented in Table 5.

The separation of precious metals such as platinum-group metals (PGMs) or other noble metals has been one of the most studied applications of PIMs. A PIM made of poly(vinylidene fluoride-co-hexafluoropropene) (PVDF-HFP) as the base polymer and Cyphos IL 104^®^ (trihexyl(tetradecyl)phosphonium bis(2,4,4 trimethylpentyl)-phosphinate) was successfully used for the selective extraction of Au(III) for *aqua regia*-igested electronic scrap [63]. PIMs with graphene oxide were used for Au extraction from acidic solutions [69]. A PIM incorporating PVC, 2NPOE, and D2EHAG was tested for Au(III) recovery from mobile phone waste leachate. The optimized PIM extraction ensured the transport of 96% of the Au(III) from the leachate into the receiving solution [64]. A PIM with [A336][SCN] as the carrier was tested for Au(I) recovery from alkaline cyanide solutions. Over 98.2% of the Au in the feed solution was transferred through the membrane into the stripping solution [65]. Also, Zhu et al. [70] prepared a PIM created using guanidinium ionic liquids, for the selective extraction of Au(I) from cyanide-containing wastewater. Using a PIM containing calix[4]pyrrole derivative and CTA as the base polymer, Ag(I) was separated from aqueous nitrate solutions [71].

Fajar and collaborators [66,72] prepared PIMs containing PVDF-co-HFP, 2NPOE, and P 88812Cl as a carrier for the extraction of PGMs (Pt, Pd, and Rh) from spent automotive catalysts. Keskin et al. [73] prepared PIMs using different mixtures of base polymers (PVC, PVDF, CTA), carriers (Aliquat 336, trioctylamine), and plasticizers (2NPOE) and tested them for the Pd(II) extraction. In another study, the effects of membrane composition and the feed and stripping phases on Pd(II) extraction were evaluated [74].

An important element with applications in high-technology industries (batteries, magnetic materials, catalysts, etc.) is Co, and thus research was carried out on its extraction. A PIM containing CTA, D2EHAG as a carrier, and dioctylphthalate as a plasticizer was tested for the separation of Co(II) from an acidic solution containing Mn(II) [14]. Lithium is another critical element for various industries, and thus its production at a high purity has been extensively studied. A new PIM comprising green polyol as the base, 1-butyl-3-methylimidazolium chloride as the ionic liquid, and 12-Crown-4 (12C4) as the carrier was tested to extract Li^+^ from an aqueous solution [75]. Paredes et al. [67] developed a system to concentrate Li^+^ from alkaline aqueous media using a PIM composed of CTA, and LIX-54 100 and Cyanex 923 as the carriers. The system showed a much higher selectivity for Li^+^ compared to other cations in solution.

In another recent study [76], a PIM produced from CTA as the base and tributyl phosphate together with sodium tetraphenylborate as the carrier was also reported as highly selective for Li^+^. Zeng et al. [68] also prepared a PIM for Li^+^ extraction from brines, in which the carriers were tributyl phosphate and 2-ethylhexyl phosphonic acid mono 2-ethylhexyl. The authors found that Li^+^ can be stripped from the carriers using only water. Moreover, the membrane displayed good permeability and selectivity for Li^+^ extraction.

A PIM prepared from CTA as the base polymer and 1,10-Phenanthroline as the ion carrier was used for Co, Cu, and Ni from aqueous solution. The results indicated the extraction of 91% Co, 89% Ni, and 89% Cu from aqueous solution [77]. PIMs were prepared and used for the separation of nonferrous metal ions from aqueous solutions [78,79,80,81,82]. For Cu(II) extraction from ammonia solutions PIMs containing LIX84I (approx. 50 of the active component 2-hydroxy-5-nonylacetophenone oxime) as a carrier were tested with promising results [83]. The recovery of nutrients from different types of samples was another application of PIMs. For example, Casadellà et al. [84] used a PIM made from CTA, 2-NPOE (plasticizer), and dicyclohexan-18-crown-6 (DCH18C6) as a carrier for the selective recovery of K^+^ from urine.

### 4.5. Comparison of PIM Technology with Competing Metal Ion Separation Technologies

A short, critical evaluation of the possible advantages and disadvantages of PIMs in their four main domains of application is presented in Table 6.

Table 7 gives a comparison of PIM membrane technology with other metal ion separation technologies (supported liquid membranes (SLMs), ion exchange (IE), and solvent extraction (SX)) in terms of selectivity, stability, environmental impact, ease of operation, and scalability.

Thus, PIM technology offers an equilibrium between technical properties, environmental impact, and overall costs, making it a promising alternative to classical separation technologies. The main drawback in its industrial application remains its low transport rates, which can present a problem, especially in water treatment and industrial sectors.

## 5. Efficiency of Polymer Inclusion Membranes for Various Metals

Because the separation of ionic species by PIMs is based on the extractant activity, the efficiency of the membranes is mostly correlated with the extractant type. There are several ways to evaluate the separation efficiency of PIMs. However, in many cases, the efficiency of metal ion transport through the PIM is assessed by a recovery factor (RF), which accounts for the initial concentration (*Ci*) and a concentration (*C*) at time *t* in the feed solution. RF can be computed using Equation (9) [26]:

(9) $$RF=\frac{C_{i}-C}{C_{i}}\cdot 100\%$$

In feed solutions with a multielement composition, the accumulation efficiency (AE) was calculated to evaluate the competitive accumulation of these elements. Table 8 presents a summary of recent publications reporting the transport efficiencies of PIMs for various metal ions.

## 6. Components of Polymer Inclusion Membranes Obtained from Sustainable Sources

**Figure 5:**
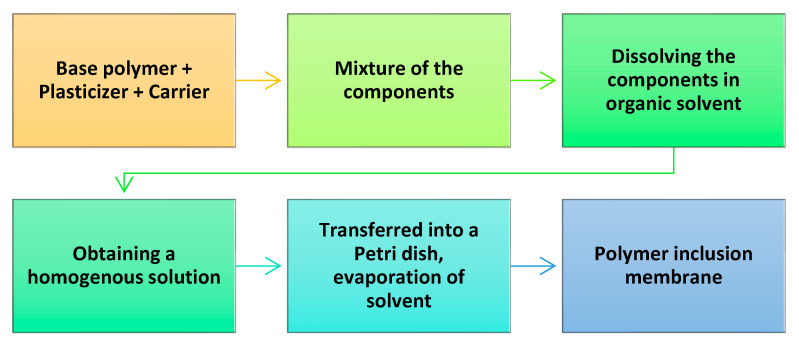
Scheme of the preparation steps required to obtain PIMs.

In order to ensure the efficient transport of metals from the feed solution, the PIMs should have several components that provide them stability, flexibility, and selectivity in extracting the target ions. Each of these main properties is provided by specific components, which need to be mixed in specific conditions. A general scheme for the production of PIMs is indicated in Figure 5 [93,94].

Usually, PIMs comprise three main components: (*a*) a base polymer, which provides support and mechanical strength ensuring stability; (*b*) a plasticizer, which enables membrane elasticity; and (*c*) a carrier, which constitutes the “core” of the membrane, providing the transport of metal ions across the membrane and selective extraction [95,96]. PIMs can be produced by dissolving all the components—the base polymer, carriers, and plasticizers—in an organic solvent. After homogenization, the solvent is evaporated to obtain the targeted membrane.

In the production of PIMs, according to the published literature, PVC and CTA have been selected as the base polymers since these are commercially available and possess well-established properties. Another advantage is that they are compatible with the most widely used carriers, such as D2EHPA and Aliquat 336. PVC is a moderately polar polymer with a low grade of crystallinity; thus, the membranes produced by casting PVC are rigid by nature, and typically a plasticizing agent is required. On the other hand, CTA is highly polar and has a high degree of crystallinity, which offers outstanding mechanical strength for the membrane [97].

An alternative to producing less waste of polymers in PIMs is the utilization of polymers with an increased mechanical strength, reusability, and transport rate. In this sense, poly(vinylidene fluoride-co-hexafluoropropylene) (PVDF HFP) has been proposed as a promising option for use as a base polymer [15]. This polymer has higher flexibility, which supports improved PIM permeability [63,95,96].

However, considering the environmental problems created by the use of polymers of petroleum origin, their replacement with polymers derived from sustainable sources is of great interest, considering their characteristics and biodegradability [98,99]. According to recent studies, biodegradable polymers represent sustainable alternatives for membrane production. Based on the ways they are obtained, biopolymers can be divided into three groups: (*a*) polymerization of monomers; (*b*) conversion by microbial fermentation; (*c*) chemical modification of natural products [99]. Among these, polylactic acid (PLA) is considered one of the emergent biopolymers that can rapidly replace petroleum-origin polymers. PLA has several features that are similar to well-known polymers such as polyvinylchloride, polypropylene, polystyrene, etc., in terms of flexural strength, elongation, yield strength, or tensile modulus. Moreover, PLA can be easily molded and reshaped in diverse forms using processes such as extrusion or injection molding [100]. In comparison with fossil-based polymers, PLA has higher permeability and a lower melting point and thermal stability, which may require its modification for some applications [101]. Surface modification of PLA expands its domains of applications by adapting its characteristics to specific requirements [102]. Also, to enhance PLA characteristics, many recent investigations have been carried out to produce PLA nanocomposites. The addition of nanoparticles reveals a notable improvement in the mechanical and thermal properties of PLA [103,104,105,106]. A major advantage of PLA over other bioplastics is its high production capacity, as it can be produced from a variety of bio-sources. Thus, this is a green and cost-effective alternative to fossil-based plastics and is commercially available [107,108].

Due to its favorable properties, PLA has been found to be suitable for the fabrication of membranes with applications in separation, water treatment, ion exchange, and adsorption [89]. Gardner et al. [109] investigated the performance of several cellulose derivative polymers such as cellulose acetate propionate (CAP), cellulose acetate butyrate (CAB), and cellulose tributyrate (CTB) in the production of PIMs encompassing bis-tert-butylcyclohexano 18-crown-6 as a carrier for K^+^ transport. The tested PIMs showed an improved performance in terms of extraction degree and robustness in acidic and alkaline environments. Kunene et al. [110] created a new PIM using polysulfone (recognized for its stability, hydrophobicity, and durability) as the base polymer and Aliquat 336 as the extractant. They reported that a growth in carrier concentration improves the membrane’s hydrophilicity and that the produced PIMs were stable up to 180 °C.

Keawsupsak et al. [111] investigated the use of PLA or biodegradable polymer combinations to produce filtration membranes. The results showed that the PLA membrane efficiently removed contaminants and improved tensile properties. Sellami et al. [112] reported that a combination of CTA and poly(butylene adipate-co-terephthalate) (PBAT) was effective in Cr(VI) separation, with efficiency improving as the proportion of PBAT was increased. Later, the same research group developed PIMs for Cr(VI) using PVDF modified by montmorillonites [48]. Scaffaro et al. [113] created a PLA with a polyethylene oxide (PEO) electrospun membrane for oil adsorption.

Recently, Hammadi et al. [25] tested a biodegradable polymer blend consisting of 54% PLA and 13% PBAT as base polymers. Aliquat 336 at a concentration of 30% was used as the ion carrier next to hybrid nanofillers such as 3% graphene oxide (GO) and/or 3% modified montmorillonite. The combination produces synergistic benefits from the addition of the modifiers, resulting in a higher extraction efficiency of Cr(VI). PIMs based on biodegradable cellulose and Algerian clay were produced and tested for metal removal from wastewater [114].

Darvishi et al. [115] developed a new PIM with cross-linked high-molecular-weight green polyol (GPO) made from castor oil as the polymer base, as an alternative to the “classical” base polymers such as PVC, PVDF, or CTA. The PIM was tested for selective extraction of Ca^2+^ over competitive ions like Na^+^, K^+^, and Mg^2+^. The tests indicated an improved selectivity and flux of Ca^2+^. To improve the PIM performance, Ershad et al. [116] proposed the use of purified dinonylnaphthalene sulfonic acid (DNNS). The PIMs obtained in this way show an improved extraction performance and better stability. A sustainable non-plasticized PIM containing an immobilizing optode ligand has been developed for in situ colorimetric determination and pre-concentration of Be^2+^ in biological and environmental samples by the encapsulation in PVC of selective ligand (E)-6-(4-((2,5-dihydroxyphenyl)diazenyl)phenyl)-2-oxo-4-phenyl-1,2-dihydropyridine-3-carbonitrile [117].

Maiphetlho et al. [118] developed new PIMs, which included silver nanoparticles (AgNPs), for the extraction of divalent cations (Cd^2+^, Co^2+^, Cu^2+^, and Ni^2+^) from contaminated water. The PIMs containing AgNPs displayed an improved transportation capacity compared to simple PIMs. In another study [70], the separation of Cd^2+^, Co^2+^, Cu^2+^, and Ni^2+^ was investigated using PIMs containing ethylenediamine-bis-acetylacetone as the carrier. Hu et al. [119] proposed a chemical modification of PIMs containing PVC, NPOE, and 2 hydroxy-5-nonylacetophenoneoxime (LIX^®^84I), using modifiers with polar groups. The PIMs were tested for Cu(II) transportation and presented significantly improved permeability and transport efficiency compared to the unmodified PIMs.

According to the literature, PIMs utilizing petroleum-based polymers such as PVC and CTA are confirmed as having a commendable stability, robustness, and extraction efficiency [120]. The shift towards sustainable materials brings environmental benefits, including a reduced carbon footprint. It has been estimated that PLA production uses around 55% less fossil energy and releases significantly less CO_2_ compared to traditional polymers [121]. However, despite their environmentally friendly features, the use of biodegradable polymers in PIMs presents some challenges. The robustness of PIMs for applications at a large scale still seems to be limited, mainly due to their degradability. However, limited data are available in the literature offering a comprehensive analysis regarding the stability and extraction efficiency between PIMs based on biodegradable polymers and those based on petroleum-based polymers, though several studies present the stability and extraction efficiency of PIMs based on biodegradable polymers, mainly PLA. This is one of the desirable biodegradable polymers because it is soluble in many organic solvents, and thus PLA-based PIMs can be prepared by the casting method. Despite some advantages of PLA membranes, their impact resistance is inferior to that of conventional polymers used for PIM fabrication.

Semicrystalline PLA has a better stability than amorphous PLA due to its higher shear viscosity and differences in macromolecular structure. Furthermore, the mechanical characteristics of PLA can be adjusted by incorporating different mixtures [107]. For instance, the incorporation of zeolites or graphene oxide in biodegradable polymers enhanced membrane strength and functionality [122]. A PIM based on a biodegradable PLA/PBAT polymer blend, filled with Cloisite 30B (C30B) and/or graphene oxide (GO) fillers and Aliquat 336 carrier, was tested for Cr(VI) transport and reached an extraction efficiency of 79.5%. Moreover, it achieved an 84.4% extraction efficiency when 3% GO was added to the PIM composition [25].

## 7. Green Solvents and Green Methods for the Preparation of PIMs

Although PIM-based metal extraction is a green alternative to classical solvent extraction because it significantly decreases the use of toxic solvents, these are still widely used in PIM production. To reduce this drawback, efforts have been made in the last years to replace toxic solvents with greener ones, or to drastically reduce or eliminate the use of solvents by improving the preparation technologies [123].

In a recent paper, the suitability of several non-toxic and “green” solvents obtained from renewable sources, such as ethyl acetate, acetone, 2-methyltetrahydrofuran, and dihydrolevoglucosenone (CyreneTM), was tested for the manufacture of PIMs comprising the most commonly used polymers (PVC, CTA, and PVDF-HFP) and extractants (Aliquat 336 and D2EHPA) [16]. The authors reported a similar extraction efficiency and membrane stability when PIMs were prepared using THF as the solvent or green solvents. Even though imaging techniques revealed changes in the surface morphology of PIMs made using classical or green solvents, the extraction performances were not substantially affected.

The scientific literature indicates that the choice of solvent in manufacturing PIMs can significantly influence their porosity, morphology, and selectivity. The use of less hazardous solvents can lead to the formation of membranes with different morphologies, often resulting in more asymmetric structures, depending on the solvent–nonsolvent exchange rates. The kinetics of phase inversion can be sluggish, leading to slower pore formation [124]. Conventional solvents (THF, chloroform) enable fast phase separation, resulting in porous membranes with a dense and uniform polymer distribution, which have a good separation efficiency. Nevertheless, the high volatility of these solvents can occasionally cause pore reduction post-fabrication, which can be a disadvantage. Green solvents can produce membranes with higher porosity than conventional solvents. For example, when dialkyl carbonates were used as solvents for PIM fabrication, the results showed that porosity tendencies can vary during the fabrication process depending on specific conditions [125]. In regard to PIM selectivity, some green solvents enhance hydrophilicity, improving antifouling properties and selectivity [126]. Consequently, the choice of solvent has a decisive role in determining the porosity, morphology, and selectivity of PIMs. Green solvents can be an alternative to classical solvents but produce membranes with a less predictable morphology and higher porosity, requiring process optimization depending on the intended use.

An alternative green approach to producing PIMs entails the elimination of solvents through the implementation of a thermal compression technique. This technique involves melting the PIM components and the subsequent application of high pressure to the molten specimen, resulting in the formation of a flat-sheet film [124]. A variety of polymers were evaluated in the study, including two cellulose derivatives and two thermoplastic polymers: polyurethane (TPU) and poly-caprolactone (PCL). The composition of PIMs also incorporated the ionic liquid Aliquat 336. The investigation revealed that TPU and PCL membranes exhibited non-oily, translucent, whitish, and flexible characteristics. In contrast, cellulose-derivative polymers resulted in films that did not meet the desired standards. Notably, PCL-based membranes exhibited enhanced stability compared to those prepared by solvent casting. Furthermore, PIMs derived from TPU and PCL membranes demonstrated a remarkable 90% extraction efficiency for the separation of Cr(VI). Other authors produced micropolymer inclusion beads (μPIBs) [127]. The μPIBs tested contained PVC or PVDF-HFP as the polymer and D2EHPA as the extractant.

To mitigate the necessity of producing a substantial quantity of PIMs, some authors have directed their attention toward the fabrication of membranes that exhibit high stability, enabling their repeated utilization. Innovative composite PIMs were prepared by a modified solvent evaporation method using a porous polytetrafluoroethylene as the base membrane, PVC polymer dibutyl phthalate as the plasticizer, and 2-ethylhexyl phosphonate mono-2-ethylhexyl ester as a carrier [128]. The obtained PIMs had a higher tensile strength (36.86 MPa to 52.77 MPa) and bursting strength (>0.2 MPa) than conventional PIMs. The authors concluded that composite PIMs could be reused multiple times due to their consistent mechanical properties and separation performance, indicating their industrial application potential.

Ouchun et al. [129] prepared PIMs from methyltrioctylammonium oleate fixed in a mixture of PVDF and polysulfone, characterized by long-term stability [104]. The inclusion of the ionic liquid with a percentage of 33% conferred the PIM a highly hydrophobic and porous character, thereby rendering it suitable for use in desalination processes. Recently, a novel hydrophobic PIM was developed for direct-contact membrane distillation (DCMD) application [130]. PIMs were obtained using PVDF loaded with the ionic liquid methyltrioctylammonium bis(2-ethylhexyl) phosphate. The resulting membrane proved to have outstanding stability, with negligible degradation over multiple cycles and prolonged operation in water desalination.

## 8. Scalability of PIM-Based Separation

PIMs have emerged as a promising technology for metal ion separation processes in a variety of applications, including water treatment, metal recovery, and tools in analytical chemistry for water quality assessment and monitoring. Nevertheless, for a successful scaling up to industrial applications from laboratory-scale research, understanding the scalability of PIMs is critical. The production cost of PIMs can vary depending on factors such as material selection and manufacturing processes. Remarkably, a recent study reported that producing 1 m^2^ of PIM may cost approximately USD 0.08 to 0.16 [33].

PVC and CTA are polymers that are commonly used and affordable. The newly tested polymeric component PLA is also a promising alternative. The common carrier agents that showed good selectivity and stability are Aliquat 336 and D2EHPA. As manufacturing techniques, transitioning from casting production to continuous roll-to-roll processing represents a solution for large-scale PIM production.

Next to PIM production costs, other expenditures associated with PIM-based processes include energy consumption [131]. Ghaffour et al. [132] calculated for membrane-base processes that energy consumption represents around 69% of the total costs, membrane replacement accounts for about 21%, while material costs constitute approximately 10% of the overall expenses. Compared to other separation processes based on pressure-driven systems such as reverse osmosis, PIM-based separation characteristically operates at a lower energy consumption. PIMs normally operate at atmospheric pressure, which reduces energy needs.

Considering the two main aspects, the cost of producing PIMs and the energy required for functioning, it can be stated that PIM-based separation technology represents a promising option for industrial-scale applications. Still, challenges in PIMs’ sustainability, stability, and large-scale production must be addressed. Future research is necessary on optimizing PIM formulations, improving manufacturing techniques, and conducting life cycle analyses to increase economic viability.

## 9. Conclusions and Future Perspectives

This review has summarized the recent literature on the developments of polymer inclusion membranes (PIMs) for metal ion separation, with a particular focus on the trend of pursuing alternative, greener PIM production. PIMs have found applicability in various fields such as analytical chemistry, water quality monitoring, water treatment, and metal recovery. PIMs typically include a base polymer, a carrier, and, if necessary, a plasticizer. PIMs can be formed by dissolving all the components—base polymer, carriers, and plasticizers—in an appropriate solvent. After homogenization, the solvent is evaporated to achieve the desired membrane. Consequently, numerous studies have been carried out to develop PIMs tailored toward specific analytes and specific matrices, and various authors have contributed to the improvement of PIMs by including new materials for these components.

Although PVC and CTA continue to be the most commonly used base polymers in PIMs due to their wide availability, stability, and compatibility with an extensive range of plasticizers and carriers, they are of petroleum origin, which can cause environmental issues. A possible substitute to produce less polymer waste is to use polymers with a better mechanical strength, reusability, and transport rate, and, in this respect, PVDF HFP has been reported as a promising option. Moreover, biodegradable polymers obtained from sustainable sources are increasingly studied for membrane production. Thus, it is expected that future studies will focus on the replacement of petroleum-based polymers with biodegradable and sustainable sources.

The carriers ensure the metal ion transport across the membrane and thus are the main component responsible for PIM selectivity and the transportation rate. Among these, Aliquat 336 and D2EHPA have been the most studied, but on this topic, many more alternatives have also been studied to ensure selectivity towards target metals. Derivates of pyridine, calix[4]arene, and Kelex 100 were among the carriers tested in recent studies. However, further research is required to find novel carriers with improved characteristics.

The plasticizer is mainly used to provide membrane elasticity, and in some cases, it is not included in the PIM composition. NPPE and NPOE were largely used as plasticizers in the studies reported in the literature. Since the number of known plasticizers is still limited, future research should be conducted to discover new appropriate types of plasticizers.

One of the drawbacks of PIM fabrication is the need to use volatile and toxic organic solvents. To avoid this, several papers present the possibility of their replacement by greener solvents or to produce PIMs using solvent-free methods. This is clearly a topic of interest for future research.

Nevertheless, challenges in PIMs’ sustainability, stability, and large-scale production must be addressed. Future research is necessary to optimize PIM formulations, improve manufacturing techniques, and conduct life cycle analyses to increase economic viability. While specific studies focusing exclusively on PIMs under real wastewater conditions are limited, insights from broader research on polymeric membranes provide valuable guidance. Enhancing biofouling resistance through material modifications and understanding the impacts of fouling and cleaning on long-term performance are critical steps. Future research should prioritize long-term, real-world testing of PIMs to validate their practical utility in wastewater treatment applications.

PIMs can be used as a passive sampling tool, as they are well-integrated in the trend in analytical chemistry of working toward greener sample preparation. This can improve the performance parameters of analytical methods, reduce the chemical and physical interferences in metal determination in complex matrices, and reduce the number of sample preparation steps. In this field of research, the production of membranes capable of extracting more components simultaneously may be a future development. Passive sampling is also a useful instrument for mimicking metal bioavailability in soil and evaluating the uptake by crops. Future studies linking PIM separation with plant bioaccumulation will provide a more complete understanding of metals’ bioavailability and mobility in soil.

## Figures and Tables

**Table 1:** Examples of initial maximum fluxes obtained for PIMs prepared using different carriers [21].

Metal Ion	Carrier	*J_M_* × 10^10^ (mol/cm^2^ s)
Cd(II)	TOPO	2.924–3.036
Zn(II)		3.332–3.477
Pb(II)		0.2342–0.2485
Cd(II)	Aliquant 336	3.345–3.718
Zn(II)		1.473–1.680
Pb(II)		0.3340–0.23424
Cd(II)	Cyphos IL 101	3.067–3.241
Zn(II)		2.190–2.307
Pb(II)		0.7000–0.7189
Cu(II)		0.006916–0.01110
Cd(II)	D2EHPA	0.4548–0.4684
Zn(II)		3.413–3.527
Pb(II)		2.747–2.881
Cu(II)		0.2121–0.2425
Cd(II)	RILC8_Br	7.260–9.190
Zn(II)		1.345–2.414
Pb(II)		1.049–1.344

**Table 2:** Examples of PIMs’ uses for metal ion determination in chemical analysis.

Analytes	Type of Sample	Characteristics of PIMs	PIMs Utilization	Refs.
As(III) and As(V)	As-contaminated water	50% CTA and 50% Cyanex 301, (bis(2,4,4-trimethylpentyl) dithiophosphinic acid)	As(III) and As(V) speciation. As(III) extracted on PIM determined energy-dispersive X-ray fluorescence (EDXRF) spectrometry	[31]
As(III) and As(V)	Groundwater	Polyvinyl chloride (PVC) and 20% or 31% Aliquat 336	As(III) and As(V) speciation. If As(III) is oxidized to As(V), total As is preconcentrated	[32]
As(III) and As(V)	Water	50% CTA and 50% Aliquat 336	As(III) and As(V) speciation. The produced PIM allowed As(III) and As(V) separation and preconcentration	[33]
As(V)	Water	PVC/RPVC + benzalkonium chloride (BAC)	As(V) extraction and preconcentration at pH = 7, separation from complex matrix	[34]
As(V)	Drinking water	Poly(vinylidenefluoride-co hexafluoropropylene) and Aliquat 336	PIM included in flow analysis manifold for As(V) for separation, preconcentration, and detection using hydride generation	[35]
Pb(II), Cd(II), and Zn(II)	Seawater	CTA + Aliquat 336 or di-(2-ethylhexyl) phosphoric acid (D2EHPA) as carriers + 2-nitrophenyl octyl ether (NPOE) as plasticizer	Selective separation of Pb(II), Cd(II), and Zn(II) from complex matrix of seawater	[36]
Hg	Natural waters	CTA + thiosalicylate (TOMATS) or salicylate (TOMAS) as carriers + NPOE	Hg extraction and preconcentration in natural waters	[37]
Zn(II)	Nutrient solution, hydroponic media	70% CTA as polymer + 30% D2EHPA as carrier; nitric acid 0.01 M receiving solution	Evaluation of Zn bioavailability to potato plants, by measurement of PIM-device fluxes of Zn(II)	[38]
Zn(II) and Cu(II)	Natural waters	CTA + D2EHPA + NPOE; different composition nitric acid 0.01 M receiving solution	PIM used as sensor to assess Zn and Cu complexation	[39]
Cd(II)	Aqueous solution, water	CTA + Kelex 100 + NPOE	PIM used as sensor for Cd by in situ visible and mid-Fourier transform infrared spectroscopy	[40]

**Table 3:** Summary of relevant studies of PIMs as passive samplers for metal ions.

Analyte	Type of Sample	Characteristics of PIMs	Uses of PIM-Based PS	Refs.
Zn(II)	Urban pond waters	60% PVC (base) + 40% D2EHPA (carrier)	Two approaches used: immersion in feed solution and feed solution flown through the membrane	[23]
Zn(II)	Urban pond waters	50% CTA (base) + 40% NPOE (plasticizer) + 30% Cyphos104 (carrier)	A system containing a PIM to mimic biofilm zinc accumulation in polluted mine stream water	[43]
Cu(II)	Aqueous solution	CTA (base) + Tris-(2-ethylhexyl) phosphate TEHP (plasticizer) + Kelex-100 (carrier), 38 cm membranes	Experiments carried out in a homemade device resulted in a calibration equation for the estimation of the Cu(II) concentration in aqueous solutions	[44]
Cu, Ni, Co, Cd	Surface water	(60–90%) PVC (base) + (10–40%) D2EHPA (carrier)	A polytetrafluoroethylene (PTFE) that has a lumen receiver (5.5 mL) solution separated by a PIM from the source	[45]
Zn(II)	Freshwater	PVC + dinonylnaphthalene sulfonic acid (DNNS) or D2EHPA, +1-tetradecanol (modifier)	A flow-through passive sampler (FTPS) composed of 3 glass vessels each attached to a flow-through compartment	[46]

**Table 4:** Summary of recent studies of PIMs for toxic metal ion removal from water.

Analytes	Type of Sample	Characteristics of PIMs	Outcomes	Refs.
Hg(II)	Industrial wastewater	CTA (base) + 2-NPOE (plasticizer) + calix [4]pyrrole derivative (carrier)	Separation efficacy of 91.8% for Hg(II) ion removal from wastewater	[47]
Cr(VI)	Chrome plating bathwater	CTA (base) + 2-NPOE (plasticizer) + *p*-*tert*-butylcalix[4]arene amine derivative (carrier)	Transport efficiency about 97.69%	[26]
Cr(VI)	Polluted water	PVDF (base) + Aliquat 336 (carrier) + Montmorillonite	Permeation flux up to 2.7 mol/(m^2^ s)	[48]
Cr(VI)	Aqueous solution	Poly(ethylene-co-vinyl acetate) (EVA) (base) + Aliquat 336 (carrier)	Transport flux up to 54.7 µmol/(m^2^ s)	[49]
Cr(III), Zn(II), and Ni(II)	Post-galvanic wastewater	PVC + 3-propyl-pentane-2,4-dione (carrier)	62–64% of Cr(III) and 75–78% of Zn(II) can be recovered using membrane technique	[50]
As(V)	Acid mine drainage (AMD)	CTA (base) + 2-NPOE (plasticizer) + Cyanex 921 (carrier)	Separation efficiency of 90% for As(V) removal from AMD	[51]
Ni(II)	Aqueous solution	CTA (base) + TEPH (plasticizer) + Kelex 100 (carrier)	96% of Ni(II) was transferred in the receiving solution	[52]
Cu(II)	Aqueous solution	CTA (base) + 2-NPOE (plasticizer) + 1-alkyl-1,2,4-triazole (carrier)	Flow rate across the membrane in the range of 16.1 mol/(m^2^s) to 1.59 mol/(m^2^s)	[53]
Cd(II), Co(II), Cu(II), Ni(II)	AMD	60% PVC (base) + 40% D2EHPA (carrier)	Accumulation of 63.42 mg/L Ni, 57.34 mg/L Co, 49.86 mg/L Cu, and 47.48 mg/L Cd	[54]

**Table 5:** Selection of recent studies related to PIMs for valuable metal ion recovery from wastes.

Analytes	Source Materials	Characteristics of PIMs	Outcomes	Refs.
Au(III)	Acid-digested electronic scrap	Poly (vinylidene fluoride-co-hexafluoropropene) (PVDF-HFP) + Cyphos IL 104^®^	Selective transport of Au(III) from digested electronic scrap in aqua regia	[63]
Au(III)	Mobile phone leachate	PVC + 2NPOE + D2EHAG	96% of the Au(III) was selectively recovered into the receiving solution	[64]
Au(I)	Alkaline cyanide solutions	PVC + 2NPOE + [A336][SCN] (carrier)	More than 98.2% of extracted into the stripping solution	[65]
Pt(IV), Pd(II), Rh(III)	Spent automotive catalysts	PVDF-co-HFP + 2NPOE + Trioctyl (dodecyl) phosphonium chloride (P 88812 Cl)	Pd(II) with a very high purity (close to 100%), Pt(IV) recovery 90%	[66]
Co(II)	Feed solution containing Co(II) and Mn(II)	CTA + dicyclohexan-18-crown-6 (DCH18C6) + 2-NPOE	Complete transfer of Co(II) from feed solution and <5% Mn(II) transferred	[14]
Li(I)	Alkaline aqueous media	CTA + LIX-54 100 and Cyanex 923 as carriers	Increased selectivity for Li^+^ compared to other cations in solution	[67]
Li(I)	Brines	CTA + 2-ethylhexyl phosphonic acid mono 2-ethylhexyl (P507) and tributyl phosphate	Li^+^ was stripped from the extracting carriers using water without the addition of HCl	[68]

**Table 6:** The advantages and disadvantages of PIMs in their four main domains of application.

Domain of Application	Advantages	Disadvantages
Chemical analysis	Ability to perform extraction and back-extraction at the same time Reduced quantity of solvents used for sample preparation Analyte extraction in a simple matrix, reducing interferences Analyte preconcentration decreases LOD/LOQ High selectivity toward analytes, with applications in chemical speciation Component of ion-selective electrodes or optodes	Not yet commercially available Low applicability for multielement sample preparation Different PIM components give different diffusion coefficients, or mass transport, which should be tested before use for real sample analysis Needs extended validation studies to assess the performance parameters
Water monitoring	Applicability in passive sampling with all advantages, such as no need for energy sources for sampling, and time-averaged concentrations over the deployment period On-site preconcentration, meaning no need to handle large volumes of water On-site chemical speciation, avoiding changes in chemical speciation from sampling to laboratory analysis Simplicity in construction, raising the possibility of inclusion in portable devices Compared to other PS techniques, like DGT, there is no need for an elution step Applicability for bioavailability studies	Not yet commercially available (as in other PS techniques like DGT) Low applicability for multielement monitoring Like in laboratory chemical analysis, different PIM components give different diffusion coefficients, which should be tested before use for real samples The possible variations in concentrations over the sampler deployment period are averaged, so that extremes cannot be detected Designed for long-period deployment, which is a disadvantage when compared with grab sampling
Water purification	High efficiency and selectivity Low energy consumption Usability at ambient temperatures No spent filtering material resulted, thus avoiding related management concerns No sludges result The metal ions removed from the treated water can be recovered Can work in waters with a low pH	Scalability to industrial needs future research Slow transport kinetics Carrier leaching from the membrane can occur High specificity can be a disadvantage for waters contaminated by multiple elements Membrane degradation in time
Circular economy	One-step process of extraction and stripping, saving time Some PIMs showed selectivity towards valuable metals Lower environmental footprint due to low amounts of solvents necessary Can be included in experimental setups for metals transport to specific electrodes The high recovery rate from solutions with complex matrices High-purity (>95%) metals can be obtained	Scalability to industrial needs future research Slow transport kinetics Membrane degradation in time Limited reuse stability The stability of PIM depends on the solubility of the ionic liquid in the feed phase The hydrophilic/hydrophobic equilibrium and the chemical nature of the carrier should be carefully selected to provide PIM stability

**Table 7:** Comparison of PIM membrane technology with other metal ion separation technologies (liquid membranes (LMs), supported liquid membranes (SLMs), ion exchange (IE), and solvent extraction (SX)).

Characteristics	PIMs	SLMs	IE	SX
**Selectivity**	High selectivity due to tailored base polymers and carriers	High selectivity, but are susceptible to carrier leakage	High selectivity, but needs regular regeneration	High selectivity, but needs large volumes of solvents
**Stability**	More stable than SLMs as it contains a solid base polymer	Relatively short functioning lifetimes	Stable, but susceptible to fouling	Necessitates phase separation stages
**Environmental impact**	Low environmental impact in the operation stage	Includes organic carriers susceptible to leakage	Generates secondary waste in the form of regenerants	Requires high volumes of organic volatile and toxic solvents
**Scalability**	Scalable due to mechanical resistance, but has low transport kinetics	Low scalability because of membrane instability	Validated in industrial applications	Regularly used in various industries, even if they have a negative environmental impact
**Costs**	Moderate costs for production, low costs for operation	Low costs for production, but low usability, increasing the total costs	High costs for production and operation	High solvent consumption, increasing the total costs

**Table 8:** Examples of transport efficiencies of different metal ions through PIMs.

Analyte	PIM Composition	Transport Efficiency	Refs.
Cr(VI)	CTA + 2NPOE + calix[4]arene	Recovery factor 97.69%	[26]
Hg(II)	CTA + 2-NPOE + calix[4]pyrrole	Hg(II) extraction efficiency ~92% for model solution and ~86% for wastewater	[47]
Cr(III), Zn(II), and Ni(II)	PVC + 3-propyl-pentane-2,4-dione	Removal efficiencies of 75–78% Zn(II) and 62–64% of Cr(III)	[50]
As(V)	CTA + 2-NPOE + Cyanex 921	Extraction efficiency ~96% from model solution and ~90% from AMD	[51]
Zn(II), Cu(II), Cr(III) and Ni(II)	PVC + acetylacetone + DEHA	Extraction coefficients of Zn(II), Cu(II), Cr(III), and Ni(II): 94%, 78%, 50%, and 9%	[85]
V(V)	PVC + oleic acid + Aliquant 336	Extraction efficiency 73% form a mono-element, 71% from a multicomponent solution	[86]
Bi(III)	PVC + D2EHPA	99% form a mono-element, 98% to 61% form a multicomponent solution	[87]
Pd(II), Ag(I), Pt(II) and Au(III)	PVC + N,N′-bis(salicylidene)ethylenediamine (carrier)	Sorption percentages if 93.23% for Ag(I), ~75% for Au(III), 69% for Pd(II), and 66% for Pt(II)	[88]
Ni(II), Cu(II), and Zn(II)	PVC + N,N′-bis(salicylidene)ethylenediamine (carrier)	Extraction efficiency of Cu(II) from ~99% to ~67%	[89]
Ni(II), Zn(II), Co(II), Cu(II), Sn(II), Pb(II), Ag(I), Pd(II), Au(III)	PVC + bis(2-ethylhexyl)adipate + Cyphos IL 101	Recovery of ~64% of Pd(II)), ~79% of Ag(I) and of ~99% Au(III),	[90]
Bi(III)	CTA + TOA + 2-NPOE/TBP/DBP/TEHP	Extraction efficiency > 97%	[91]
Cu(II)	D2EHPA + lignin	Transport efficiency 74%	[92]

## Data Availability

Not applicable.

## Abbreviations

The following abbreviations are used in this manuscript:

PIM	Polymer inclusion membrane
SPM	Solvent Polymeric Membrane
PVC	Poly (vinyl chloride)
CTA	Cellulose triacetate
PVDF	Poly(vinylidene fluoride)
PVDF HFP	Poly(vinylidene fluoride-co-hexafluoropropylene)
LOD	Limit of detection
LOQ	Limit of quantification
ISEs	Ion-selective electrodes
Cyanex 301	Bis(2,4,4-trimethylpentyl) dithiophosphinic acid
Aliquat 336	Tricaprylmethylammonium chloride
BAC	Benzalkonium chloride
NPOE	2-Nitrophenyl octyl ether
TOMATS	Thiosalicylate
TOMAS	Salicylate
D2EHPA	Bis(2-ethylhexyl) phosphate
EDTA	ethylenediamine tetraacetic acid
TEHP	Tris-(2-ethylhexyl) phosphate
DNNS	Dinonylnaphthalene sulfonic acid
EVA	Poly(ethylene-co-vinyl acetate)
Cyanex 921	Tri-noctylphosphine oxide
Cyanex 923	Trialkylphosphine oxides
Cyphos IL 104	Trihexyl(tetradecyl)phosphonium-(2,4,4-trimethylpentyl)phosphinate
P 88812 Cl	Trioctyl (dodecyl) phosphonium chloride
[A336][SCN]	Methyl trioctyl ammonium thiocyanat
DCH18C6	Dicyclohexan-18-crown-6
P507	2-Ethylhexyl phosphonic acid mono 2-ethylhexyl
LIX84I	50% active component 2-hydroxy-5-nonylacetophenone oxime
PLA	Polylactic acid
CAP	Cellulose acetate propionate
CAB	Cellulose acetate butyrate
CTB	Cellulose tributyrate
PBAT	Poly(butylene adipate-co-terephthalate)
GO	Graphene oxide
AgNPs	Silver nanoparticles
LIX^®^84I	2 Hydroxy-5-nonylacetophenoneoxime
CyreneTM	Dihydrolevoglucosenone
TPU	Polyurethane
PCL	Poly-caprolactone
μPIBs	Micropolymer inclusion beads
DCMD	Direct-contact membrane distillation
PS	Passive sampling
FAAS	Flame atomic absorption spectrometry
EDXRF	Energy-dispersive X-ray fluorescence
ICP-MS	Inductively coupled plasma mass spectrometry
